# Factors influencing variation in implementation outcomes of the redesigned community health fund in the Dodoma region of Tanzania: a mixed-methods study

**DOI:** 10.1186/s12889-020-10013-y

**Published:** 2021-01-02

**Authors:** Albino Kalolo, Lara Gautier, Ralf Radermacher, Siddharth Srivastava, Menoris Meshack, Manuela De Allegri

**Affiliations:** 1 Department of Public Health, St. Francis University College of Health and Allied Sciences, P.O. Box 175, Ifakara, Tanzania; 2 Heidelberg Institute of Global Health, Medical Faculty and University Hospital, Heidelberg University, Heidelberg, Germany; 3 Department of Sociology, Faculty of Arts, McGill University, Montreal, Canada; 4 Deutsche Gesellschaft für Internationale Zusammenarbeit, 10/319, Mtendere Drive, Lilongwe, Malawi; 5 Swiss Tropical and Public Health Institute, Socinstrasse 57, P.O. Box CH-4002, Basel, Switzerland; 6 University of Basel, Petersplatz 1, P. O. Box 4001, Basel, Switzerland; 7 Health Promotion and System Strengthening (HPSS) Project, P.O Box 29, Dodoma, Tanzania

**Keywords:** Micro health insurance, Redesigned community health fund, Implementation outcomes, Contextual factors, Tanzania

## Abstract

**Introduction:**

Micro-health insurance (MHI) has been identified as a possible interim solution to foster progress towards Universal Health Coverage (UHC) in low- and middle- income countries (LMICs). Still, MHI schemes suffer from chronically low penetration rates, especially in sub-Saharan Africa. Initiatives to promote and sustain enrolment have yielded limited effect, yet little effort has been channelled towards understanding how such initiatives are implemented. We aimed to fill this gap in knowledge by examining heterogeneity in implementation outcomes and their moderating factors within the context of the Redesigned Community Health Fund in the Dodoma region in Tanzania.

**Methods:**

We adopted a mixed-methods design to examine implementation outcomes, defined as adoption and fidelity of implementation (FOI) as well as their moderating factors. A survey questionnaire collected individual level data and a document review checklist and in-depth interview guide collected district level data. We relied on descriptive statistics, a chi square test and thematic analysis to analyse our data.

**Results:**

A review of district level data revealed high adoption (78%) and FOI (77%) supported also by qualitative interviews. In contrast, survey participants reported relatively low adoption (55%) and FOI (58%). Heterogeneity in adoption and FOI was observed across the districts and was attributed to organisational weakness or strengths, communication and facilitation strategies, resource availability (fiscal capacity, human resources and materials), reward systems, the number of stakeholders, leadership engagement, and implementer’s skills.

At an individual level, heterogeneity in adoption and FOI of scheme components was explained by the survey participant’s level of education, occupation, years of stay in the district and duration of working in the scheme. For example, the adoption of job description was statistically associated with occupation (*p* = 0.001) and wworking in the scheme for more than 20 months had marginal significant association with FOI (*p* = 0.04).

**Conclusion:**

The study demonstrates that assessing the implementation processes helps to detect implementation weaknesses and therefore address such weaknesses as the interventions are implemented or rolled out to other settings. Attention to contextual and individual implementer elements should be paid in advance to adjust implementation strategies and ensure greater adoption and fidelity of implementation.

**Supplementary Information:**

The online version contains supplementary material available at 10.1186/s12889-020-10013-y.

## Introduction

Micro-health insurance (MHI) has been identified as a possible solution to foster progress towards Universal Health Coverage (UHC) in low- and middle-income countries (LMICs). In these settings, a large reliance on the informal sector, combined with a lack of institutional capacity to collect taxes, make it difficult to envision the rapid development of social health insurance schemes. MHI schemes have been shown to improve access to care, to contribute to reducing the risk of facing catastrophic health spending due to ill health and to improve the understanding and willingness to pay for insurance [1–8]. Still, MHI schemes suffer from chronically low penetration rates, especially in sub-Saharan Africa [1, 9–15]. In turn, low penetration rates translate into a limited capacity to act as an effective strategy to expand social health protection.

A number of initiatives have been implemented to promote enrolment, with the expectation of increasing MHI penetration rates. These initiatives have generally been observed to yield a limited impact on enrolment. Interestingly, however, evaluation efforts have focused almost exclusively on the effect on enrolment [9, 10, 14, 16], while limited attention has been channelled towards understanding how such initiatives have been implemented [16–19]. This means that, at the moment, the available evidence makes it impossible to discern if the inability of these innovations to generate higher enrolment rates is because of their design or because of their poor adoption or lack of fidelity during the implementation phase.

The implementation science literature has repeatedly shown that the successful implementation of innovative interventions defined in terms of acceptability, adoption and fidelity is essential to deliver the required benefits to target populations [20–23]. At the same time, the literature has also indicated that suboptimal implementation or a failure to implement innovations as originally planned is not uncommon [24–27]. Departures from original design and weaknesses in implementation have been attributed to multiple factors, and more specifically to the context within which the intervention took place. Existing evidence indicates that a variety of factors influence the implementation processes of the interventions [28–31]. The most frequently reported factors include: (1) organizational weaknesses manifested as being blind to the advantages of external innovations, being trapped into doing business according to usual organizational models, rigidity within the organization and a lack of expertise to accommodate the innovations [32–35]; (2) political factors [20, 21, 24]; (3) cultural factors [24, 25, 32], such as belief and value systems; (4) social economic factors [22, 36], exemplified by poverty levels in the communities; and (5) stakeholders’ responsiveness [24, 25, 27], manifested as support or resistance to scheme processes. Even when the organization is ready to adopt and implement innovation, users’ perceptions and implementers’ attitudes affect the implementation process, bearing important consequences on the adaptation and adoption processes [24, 25, 32].

Furthermore, paying attention to the implementation continuum, i.e., from adoption, through implementation to sustainability of the intervention processes is considered vital in understanding the routes for the successful delivery of any intervention, including innovations [19]. As such, weakness at any level of the continuum may lead to a suboptimal delivery or a failure of the intervention. Implementation research that looks at implementation processes and at factors shaping these processes offers an opportunity to enhance a detailed understanding of the vital elements leading to implementation success or weakness and sheds light on the reasons for intervention outcomes, thus differentiating design failure from implementation failure [24, 25, 33].

The implementation of MHI schemes and of the innovations they entail comes with challenges. Understanding the factors that influence implementation may help to harness the benefits of these schemes, as problems in implementation will be addressed optimally.

We aimed to fill the existing gap in knowledge by focusingour evaluation effort specifically on implementation outcomes. Our ambition was to understand variation in implementation outcomes in relation to the factors that mediate the implementation of an MHI-specific intervention. We focused on the case of the redesigned Community Health Fund (Redesigned CHF), currently known as “Improved Community Health Fund”, also known locally as “*CHF Iliyoboreshwa*” in Tanzania, an innovative intervention launched to reform the original CHF in terms introducing; purchaser-provider split, use of insurance management system (IMIS), expanded benefit package and social marketing strategies [19, 37]. Specifically, we employed a mixed-methods design to answer the following research question: What are the implementation outcomes and their moderators within the context of the redesigned CHF program in the Dodoma region?

## Methods

### Study setting

The study was conducted in the seven districts of Dodoma region: Dodoma Municipal council, Bahi, Chamwino, Kondoa, Mpwapwa, Kongwa and Chemba. The region was selected because the CHF Iliyoboreshwa was implemented there before a wider roll out to other regions in the country. Dodoma region is the administrative capital of the United Republic of Tanzania. The region is the 8th most populous in Tanzania with a population of 2,083,588. Dodoma urban (410,956) and Chamwino (330, 543) are the most populated districts, while Bahi (221,645) and Chemba (235,711) are the least populated. Subsistence farming and livestock keeping are the main livelihood activities.

By 2015, the region had 360 health facilities providing health services [38]. Accessing health facility services was highly dependent on out of pocket payments, with little contribution from health insurance schemes. At the time of the study, about 15.9% of the households were enrolled in *CHF Iliyoboreshwa*, a rate that was considerably below what was expected by its initiators and implementers. Bahi district had the highest enrollment (25.3%), followed by Chamwino (25%), and the lowest was Dodoma Municipal council (8.6%). Implemented by the council authorities of the seven districts with support from the Health Promotion and System Strengthening (HPSS) project, the Redesigned CHF program reformed the original CHF to make it a viable social health insurance scheme [15]. The program, described in detail elsewhere [15, 37], comprised reforms across administrative, governance, and health service provision structures and management of enrolment outcomes. In the reformed scheme, the CHF management teams (comprising CHF manager, accountant, Medical advisor, District Medical officer, District planning officer, District Treasurer and District Community Development officer) were in charge of managing the scheme processes, and the council health management team (CHMT) under the leadership of the District Medical officer were in charge of providing health services to the CHF clients. The district leaders, such as the District commissioner, The District chairperson (Mayor), District Executive Director and Councillors oversaw the implementation of the scheme in their areas of jurisdiction.

### Study design

This paper is part of a broader study [19] that employed a concurrent mixed methods design [39] to assess the implementation processes of the Redesigned CHF at different levels of the district health system structures. The Good Reporting of A Mixed Methods Study (GRAMMS) framework for reporting mixed methods studies were followed in reporting the results for this study [40] (additional file 1). In this paper, we focus specifically on implementation processes at the district level.

We adopted a mixed methods approach combining quantitative and qualitative methods of data collection and analysis to provide a more comprehensive picture of adoption, fidelity and their modifiers. Based on the theory of intervention (TOI) developed by our team and described in detail elsewhere [19], we mapped the intervention district-level components to be followed up in the implementation assessment and grouped them into the following functions to facilitate analysis: 1) recruitment and training of program actors; (2) materials for the program; 3) remuneration of actors in the program; 4) monitoring progress; 5) addressing CHF benefits; 6) promotion to attract enrolments; and 7) addressing the quality of healthcare.

The quantitative component uses a multi-method approach employing a survey questionnaire and a document review checklist both aiming to identify to what extent certain processes were adopted, carried out as planned and maintained as routine processes of the scheme and the influence of moderating factors.

### Study sample

#### Sample for the quantitative component

The quantitative component of the study relied on a structured survey and a document review. The sample for the structured survey was drawn from district level implementers (*N* = 68), meaning the district CHF management team, district health management team and policy leaders (such as District commissioner, District executive director). The questionnaire was filled by all (census) concerned implementers.

The sample for document review checklist was drawn from documents from all seven districts in the region (*N* = 7). The documents that provided data for the study are: (i) program reports; (ii) day to day communication about the program i.e., letters, memos and meeting minutes; (iii) strategic and operational plans; (iv) redesigned CHF policy documents and by-laws; and (v) information from insurance information management system (IMIS) database.

#### Sample for the qualitative component

The qualitative sampling procedure was multilevel in nature, whereby the sample comprised 39 key informants purposively selected at regional and at district level. Participants ranged from regional leaders, technical advisors for the CHF, district leaders (public administrators and elected leaders), to frontline implementers (CHF officers and enrolment officers).

The respondents at regional level were selected based on their capacity and position to provide information related to the support given to the districts in implementing the CHF scheme, but also their knowledge on contextual influencers of the implementation process. The respondents at the regional level included the regional secretariat members (*N* = 7) and the development partners operating at regional level (*N* = 6).

Respondents from district levels were selected based on their experiences with the redesigned CHF, their position in the scheme and the expected knowledge they had about the scheme processes. They came from three districts: D3, D4 and D6, which had high, average and low enrolment rates respectively, in relation to the baseline values since the start of the CHF Iliyoboreshwa. The respondents at the district level included district leaders (*N* = 8), such as District commissioner and frontline implementers (*N* = 18) such as CHF manager, accountant and CHF officers. The frontline implementers were included in this part of the study to verify the information provided at district level in terms of the support provided to the village level actors.

Table 1 provides a summary of the sample size for the quantitative and qualitative sources of data.

**Table 1:** Sample sizes for the study

Study component	Type of participants	sample size (N)
***Quantitative***		
1.Questionaire	District level implementers	68
2.Document review	District documents	7
***Qualitative***		
indepth interviews to key informants	Regional secretariat members	7
	Development partner representatives	6
	District leaders	8
	District level implementers	18

### Data collection instruments and procedures

The first author managed data collection directly, with support from a small team of trained research assistants who were recruited based on their level of training (mostly bachelor’s and master’s students in sociology, anthropology, public health, development studies and rural development) and their experiences in conducting public health research.

### Quantitative data

#### Questionnaire

We used a questionnaire (additional file 2) to collect data related to implementation outcomes, focusing mainly on the adoption and FOI of the program components, such as the recruitment and the training of program actors and materials for the program as reflected in our TOI. The questions on the adoption of the scheme focused on the functionality of Redesigned CHF structures and the usability of the guidelines in daily routines. FOI questions captured adherence to or departures from the CHF standard operating procedures (CHF-SOP) and the probable related moderating factors. The questionnaire was composed of yes/no, and Likert scale questions. To ensure validity and reliability of our questionnaire, 1) Development of the questionnaire was based on our theory of intervention 2) The questionnaire was discussed among the authors to agree on how the questions are constructed to improve the face validity 3) The questionnaire by the authors together with the trained research assistants 3) Principal component analysis was employed to ascertain key constructs and come up with scales and subscales 4) Cronbach’s alphas of each of the formulated scales were determined as an indication of consistency within the scales. Cronbach’s alpha of at least 0.7 was considered acceptable to measure the internal consistency of each of the scales and sub-scales [41].

#### Document review checklist

We used a checklist to extract data from the documents relevant to the scheme adoption and implementation (additional file 3). The checklist aimed at extracting numerical information that could quantify districts’ adoption and FOI based on the redesigned CHF original plan.

### Qualitative data

We used a semi-structured in-depth interview guide to collect qualitative data from the participants. The interview guide aimed to collect experiences and opinion related to the implementation processes and the contextual influencers at the district level (see additional file 4). The interviews were conducted in Kiswahili, recorded, verbatim transcribed and translated to English by professional translators. The first author checked all the transcribed translations for accuracy.

### Data analysis

#### Quantitative analysis

##### Data from the survey questionnaire

To understand the implementation outcomes and their moderators as reported by participants in the survey we had first to define and measure the outcome and explanatory variables before proceeding to statistical analyses. The outcome variables included in our study were: adoption and FOI.

In line with our previous work [19], we measured adoption as the presence or absence of and functioning of the redesigned CHF structures, availability status and use of guidelines and related processes, and job description. The structures considered include CHF governing board, CHF management teams and the conduct of the scheduled or ad hoc meetings of these structures.

We measured FOI as adherence to the program model as originally stipulated in the Redesigned CHF standard operating procedures (CHF-SOP) and related operational document, such as planning manuals, supervision guidelines (movement plans) and financial management guidelines. To measure FOI we assessed adherence to procedures related to the seven functions of the redesigned CHF reflected in our TOI.

The questionnaire consisted of several items related to each of the outcome variables (adoption and FOI). To provide a clear picture about larger theoretical constructs under study and not to focus on single items included in the questionnaire, not meaningful in and out of themselves, composite indices were calculated based on an unweighted sum of scores of the items. Items with high face validity that measure the same construct were selected [42]. Thereafter a composite score (sub-scale) or index was obtained from the sum scores of the items. Sub-scales related to adoption were adoption of CHF structures, adoption of CHF guidelines, and adoption of the CHF processes and adoption of job-description.

Subscales related to FOI (measured as adherence) were 1) recruitment and training; (2) materials for the program; 3) remuneration of actors; 4) monitoring progress; 5) addressing CHF benefits; 6) promotion to attract enrolments; and 7) addressing the quality of health care.

It is worth to note that overall indices (composite scores) for adoption and FOI were obtained as sum scores of the sub-scales related to the adoption and FOI respectively.

Thereafter, using the median as a cut off point, adoption and FOI indices were categorized into high and low scores.

Explanatory variables here also called “modifiers of the implementation outcomes” were socio-demographic factors (Age, categorized into <=40 years and >  40 year, Sex categorized as male and female, Occupation categorized as CHF service providers, CHF fund managers and others, Level of education categorized as secondary and below and post secondary, and years of schooling categorized as <=15 years and >  15 years), Time worked in the scheme categorized as <= 20 months and >  20 months, and Time stayed in the district categorized as <=7 years and >  7 years.

In our statistical analyses, we were aided by STATA version 13 (STATA Corporation, College Station, TX, USA) through which both descriptive and detailed analysis techniques to determine the relationship between variables were applied.

Descriptive analysis based on frequency, percentages and measures of central tendency or dispersion (mean, median or standard deviation and range) were used to understand the situation related to the adoption and implementation of the scheme. We used a chi-square test to determine the association between implementation outcomes and modifiers and a *p*-value of less than 0.05 was considered significant. The unit of analysis were the respondents of the questionnaire.

##### Data from the document review checklist

The document review checklist consisted of several items grouped together per theoretical constructs intended to be measured. We assigned an arbitrary weight of score to each item. The weight scores were from 1 to 10 (or 10–100 in percentage). The summation of each item score lead to a composite variable, namely adoption and FOI index for adoption items and FOI items respectively. The analysis of data from the document review was mainly descriptive with an explanatory component in the qualitative part of the study. We conducted descriptive analysis based on frequency, percentages and measures of central tendency or dispersion (mean, median or standard deviation and range) to understand the situation related to the adoption and implementation of the scheme. The unit of analysis were the districts.

#### Qualitative analysis

Qualitative data analysis was carried out by two independent researchers (the first two authors) and utilized the framework method [43] assisted by the N-Vivo software (QRS-international). The framework method as described by Gale et al. [43] was applied to analyse the transcribed material. The framework method is flexible and follows step-by-step interconnected stages that guide the analytical process. Coding started after multiple reading of the transcripts. It started deductively based on predetermined theoretical constructs based on our TOI, followed by the addition of emerging codes as they emerged from the transcripts. The codes were applied to capture the adoption and fidelity of implementation of the scheme and the factors shaping these experiences also as explanatory to the district level implementation outcomes as determined in the quantitative document review checklist.

#### Integrated interpretation

The integration of quantitative and qualitative findings occurred at the interpretation level using a triangulation approach [39, 44, 45]. We investigated where the findings converged, offered complementary information, or appeared to contradict one another. Integration allowed us to develop a composite, holistic and cross-validated picture of the facts based on the results from both quantitative and qualitative datasets. This process of integration took place after the completion of data analysis and entailed identifying similarities and differences, merging the results and discussing the meaning of the integrated results across the two levels of analysis.

## Results

### Background information of the participants

We collected information from 68 participants, of which 47(69%) were males and 21(31%) were females. Of the participants, 31(47%) worked as CHF fund managers, and 25(36%) and 12(17%) worked as representatives of the Council Management team (CMT) and CHF service providers respectively. The characteristics of the entire sample are given in Table 2.

**Table 2:** Socio-demographic characteristics of the study participants (*N* = 68)

Variable name	Variable description	Frequency	Percent
**Age group**	<= 40 years	39	57
	> 40 years	29	43
**Sex**	Male	47	69
	Female	21	31
**Occupation**	CHF service provider	12	17
	CHF fund management	31	47
	Others	25	36
**Time worked with Redesigned CHF**	<= 20 months	23	33
	> 20 months	45	67
**Time stayed in the District**	<= 7 years	45	66
	> 7 years	23	44
**Level of education**	Secondary and below	17	25
	Post Secondary	51	75
**Years of schooling**	<= 15 years	31	45
	> 15 years	37	55

### Adoption of the scheme structures and processes

Findings from the survey revealed an overall adoption of the scheme components and processes to be at 55%. Adoption varied across components and processes, with adoption of guidelines scoring highly (51%) and adoption of the scheme processes scoring the lowest (38%). Respondents who were CHF providers and those who resided in the district for less than 7 years reported high adoption of 67 and 61% respectively. We also found that respondents with secondary education or lower (64%) reported higher adoption than their counterparts (Table 3).

**Table 3:** Level of adoption of the scheme and associated individual factors (*N* = 68)

Variable name and description	High adoption of CHF structures		High adoption of CHF guidelines		High adoption of CHF processes		High adoption of job description		High overall adoption	
**Variable name**										
Variable description	n (%)	*P*	n (%)	*P*	n (%)	*P*	n (%)	*P*	n (%)	*P*
**Age group**										
< = 40 years	18 (50)	0.117	21(58)	0.23	14 (38)	0.9	20 (60)	0.95	20 (55)	0.95
> 40 years	10 (31)		14(44)		12 (37)		19 (61)		18 (56)	
**Sex**										
Male	20(41)	0.89	25 (52)	0.87	19 (68)	0.7	25 (57)	0.31	28 (58)	0.56
Female	8 (40)		10 (50)		7 (35)		14(70)		10 (50)	
**Occupation**										
CHF service provider	5 (21)	0.98	20 (64)	0.11	14 (45)	0.17	25 (86)	0.001	21 (67)	0.18
CHF fund management	13 (65)		8(50)		6 (50)		6 (54)		6(50)	
Others	10 (40)		11 (44)		6 (24)		8 (50)		11 (44)	
**Time worked**										
< = 20 months	11 (47)	0.42	14 (61)	0.28	9 (39)	0.9	14 (63)	0.74	14 (61)	0.55
> 20 months	7 (20)		21 (47)		17 (37)		25 (59)		24 (53)	
**Time stayed in the district**										
< = 7 years	20 (44)	0.44	24 (53)	0.67	20 (44)	0.14	25 (59)	0.74	27 (60)	0.33
> 7 years	8 (35)		11 (48)		6 (26)		14 (63)		11(48)	
**Level of education**										
Secondary and below	9 (53)	0.25	8 (47)	0.67	6 (35)	0.77	12 (70)	0.34	11 (64)	0.39
Post Secondary	19 (37)		27 (53)		20 (39)		27 (57)		25 (51)	
**Years of schooling**										
< = 15 years	12 (38)	0.7	12 (38)	0.05	12 (38)	0.94	18 (62)	0.86	18 (58)	0.74
> 15 years	16 (43)		23 (62)		14 (38)		21(60)		20(54)	

N (%) number and percentage of participants in the study; *P*-values are from Pearson Chi-Square test

A review of documents revealed a full adoption of the formalization of the Redesigned CHF program in that all districts had signed a contract between them and HPSS project to implement the redesigned CHF. Also, all districts had issued a job description to all actors in the scheme. The functioning of the CHF management team was also highly adopted (82%); the location of the CHF office separated from the health department (71%) and the presence of functioning CHF boards (70.7%). The actual separation of the purchaser and provider by Redesigned CHF- Council Health Management Teams (CHMT) contracts was lowly adopted (46%). The review of document indicated the mean adoption score of the scheme to be 78.5%. The highly performing districts were D5 (95%), D2 (87%) and D1 (79%). D4 (62%) was the lowest performing district (Table 4).

**Table 4:** Scores for adoption of the scheme components in the seven districts of Dodoma

Adoption of components	D1	D2	D3	D4	D5	D6	D7	Total score per CHF component	Mean score per CHF component
Recruitment and training	70	80	70	50	80	80	70	500	71
Materials for the program	50	100	50	50	50	50	100	450	64
Remuneration	80	80	60	60	80	80	60	500	71
Monitoring progress	70	100	70	60	100	70	70	540	77
Addressing CHF benefits	100	100	100	100	100	100	100	700	100
Promotion of enrolments	80	70	80	90	70	80	80	550	78
Addressing quality of care	70	90	80	70	80	80	80	550	78
Total score	520	620	510	480	560	540	560		
Mean score per district	74	88	72	68	80	77	80		

The participants in the qualitative interviews generally held positive views on the redesigned CHF. They indicated that the scheme represents an innovative effort that seeks to address the shortcomings of the old CHF scheme. They reported that the scheme has been fully adopted and taken into implementation. The adoption of the scheme by the districts was described by the interactions between the key actors and the scheme processes such as trainings, recruitment of CHF staff and promotions to attract enrolments. They indicated how they interacted with the scheme from the introduction of the scheme as follows i) their first contact with the scheme ii) their awareness of the scheme processes, iii) a deeper understanding of the innovations in the scheme iv) full usage of the scheme processes and structures. Participants also discussed the various channels through which they got in contact with the Redesigned CHF such as stakeholder meetings, official letters, promotion campaigns and operations in the districts.

*“We received an official invitation to attend the dissemination seminar of the Redesigned CHF. Many regional and district level stakeholders participated. The seminar was convened at the regional headquarters of Dodoma region.” (IDI50, NHIF representative).*

*“The first step was to invite the regional government to receive the results of the study which showed that the former CHF needed to be improved. They received the results and recommendations for steps to be taken after the study.” (IDI 45, development partner).*

Respondents also reported variations across and within districts with some places adopting the scheme processes quicker than others.

### FOI of the scheme structures and processes

Findings from the survey revealed an overall implementation fidelity of the scheme components of 58% that varied across components, with some of the components performing high (training = 92%) and others performing low (remuneration 51%). FOI was reported to be slightly higher among respondents in the following categories: less than 40 years old (52%), males (54%), worked in the scheme for more than 20 months (55%), lived in the district for more than 20 years (52%), had lower or secondary education (61%) and less than 15 years of schooling (54%) (See Table 5).

**Table 5:** Level of fidelity of implementation (FOI) of the scheme and associated individual factors (*N* = 68)

Variable name and description	High fidelity recruitment &training		High FOI in supply of materials for the program		High FOI in monitoring progress		High FOI in remuneration of implementers		High FOI in CHF benefits		High FOI in promotion to attract enrolments		High FOI in quality of healthcare.		High FOI of the program (overall)	
**Variable name**																
Variable description	n%	*P*	n%	*p*	n%	*p*	n%	*p*	n%	*p*	n%	*p*	n%	*p*	n%	*p*
**Age group**																
< = 40 years	34 (94)	0.54	19 (52)	0.28	27 (75)	0.77	19 (52)	0.89	28 (77)	0.4	18 (50)	0.68	18 (50)	0.3	19 (52)	0.89
> 40 years	29 (90)		21 (65)		23(71)		16 (50)		22 (68)		18 (56)		20 (63)		16 (50)	
**Sex**																
Male	44 (91)	0.63	29 (60)	0.67	37 (77)	0.33	24(50)	0.71	37 (77)	0.3	28 (58)	0.16	28 (58)	0.52	26 (54)	0.49
Female	19 (95)		11(45)		13(65)		11 (55)		13 (65)		8(40)		10 (50)		9 (45)	
**Occupation**																
CHF service provider	29(93)	0.41	20 (64)	0.63	26 (93)	0.206	18 (58)	0.57	21 (67)	0.27	14 (45)	0.49	15 (48)	0.48	17 (54)	0.07
CHF fund management	12(100)		7 (58)		8 (66)		6 (50)		11 (91)		7 (58)		8(66)		9 (75)	
Others	22 (88)		13 (52)		16 (64)		11(44)		18 (72)		15 (60)		15 (60)		9 (36)	
**Time (months) worked with Redesigned CHF**																
< = 20 months	21 (91)	0.76	13(56)	0.78	18(78)	0.52	11(47)	0.66	18 (78)		11(48)	0.54	9 (39)	0.04	10 (43)	0.34
> 20 months	42 (93)		27 (60)		32(71)		24 (53)		32 (71)	0.52	25 (55)		29 (64)		25 (55)	
**Time (years) stayed in the District**																
< = 7 years	44 (97)	0.023	22 (48)	0.02	32(71)	0.52	24 (53)	0.67	34 (75)	0.59	21 (46)	0.14	26 (57)	0.66	23 (51)	0.93
> 7 years	19 (82)		18 (78)		18(78)		11 (45)		16 (73)		15 (65)		12 (52)		12 (52)	
**Level of education**																
Secondary and below	16 (94)	0.78	14(82)	0.02	14 (82)	0.34	10(58)	0.48	13 (76)	0.75	9 (52)	0.99	26 (74)	0.66	11(64)	0.21
Post Secondary	47		26 (51)		36(70)		25 (48)		37 (72)		27 (53)		12 (36)		24 (47)	
**Years of schooling**																
< = 15 years	29 (93)	0.79	24(77)	0.004	24(77)	0.5	17 (54)	0.61	25 (80)	0.22	16 (76)	0.84	8 (66)	0.39	17 (54)	0.611
> 15 years	34 (91)		16(43)		26 (70)		18 (48)		25 (67)		20 (54)		30 (61)		18 (48)	

N (%) number and percentage of participants in the study; *P*-values are from Pearson Chi-Square test

The review of documents revealed that the highly scoring districts were D2 (88%), D5 (80%), D7 (80%) and D6 (77%). The lowest scoring districts were D3 (72%) and D4 (68%) (See Table 6).

**Table 6:** Scores for FOI of the scheme components in the seven districts of Dodoma

Fidelity of implementation of components	D1	D2	D3	D4	D5	D6	D7	Total score per CHF component	Mean score per CHF component
Recruitment and training	70	80	70	50	80	80	70	500	71
Materials for the program	50	100	50	50	50	50	100	450	64
Remuneration	80	80	60	60	80	80	60	500	71
Monitoring progress	70	100	70	60	100	70	70	540	77
Addressing CHF benefits	100	100	100	100	100	100	100	700	100
Promotion of enrolments	80	70	80	90	70	80	80	550	78
Addressing quality of care	70	90	80	70	80	80	80	550	78
Total score	520	620	510	480	560	540	560		
Mean score per district	74	88	72	68	80	77	80		

Respondents in qualitative interviews reported a high adherence to scheme procedures. They also reported variations in adherence and departures from CHF-SOP across districts as described below, in relation also to the key program components.

First, there were variations in recruitment and training practices across districts. Although all of the districts had recruited and trained the workforce for CHF structures using similar trainers and training materials, some districts were faster than others. There was discontinuity of training to the new CHF structure workforce and inappropriate timing of refresher trainings. Along with the issues around discontinuity of training, the functioning of the established structures had variations, especially in terms of irregularities in CHF board meetings, turnover of enrolment officers and implicit resistance to the purchaser-provider split in some districts.

Second, despite the existing outlined procedures, remuneration of the frontline implementers and health facilities was reported to vary across districts. Commissions provided to enrolment officers per enrolled households, for example, varied across districts, with some districts providing 10% (D2 and D6) while others provided 5%. While some districts provided on-spot commission to enrolment officers upon submission of enrolment forms, others delayed the payment of the commission.

Furthermore, adherence to timely payment to health facilities was sub-optimal, with problems mainly arising from the misalignment between claims submissions and payment schedules. Not surprisingly, there were opposing opinions between the district level actors: some said that delays were with the scheme paying health facilities, whereas other said that delays were caused by health facilities submitting their claims late. Third, although there were suggestions in the CHF guidelines on how to conduct promotions to attract enrolment, adaptations (modifications) of the guidelines and innovations based on district context prevailed. Interview participants described the strategies adopted by the districts as follows: local innovations, mass media, use of influential leaders, wider stakeholders’ (CSOs and community resource persons) involvement and use of sales forces such as Information, Education and Communication (IEC) materials.

*“ … for example, of the D3 innovatively started something known as “gulio la Afya” [health care open market], where they mobilized sensitization meetings not only about CHF, but also to cover health promotion issues and provide free screening for diseases and mobile health services.” (IDI45, development partner).*

Fourth, scheme benefits emerged to contain three categories: review of CHF premium and CHF benefit package by the districts, implementation of the portability of cards and cross-district reimbursement of CHF funds across health facilities. This process was reported to have high FOI across districts, except for the cross-district reimbursement, which was not implemented in any of the districts. The reasons for not implementing the cross-district reimbursement included difficulties in claiming payments from other districts when there was no clear data to indicate how much to claim (poor data records). Furthermore, some participants stated that since the district authorities all belonged to the government, it was difficult to claim and be paid.

Fifth, problems were reported with the program materials required for both enrolment and monitoring the progress of the scheme. The reported challenges were mainly on supply and maintenance of program materials being done untimely, thus leading to ineffective use of the materials by the frontline implementers of the scheme

*““We get tools like mobile phone, battery mobile charger, forms … but with mobile phones there is a challenge mostly with the charging system due to a lack of electricity in our villages, so it is would be better if when they brought bring chargers they should bring with a solar charging system (currently we have chargers that use electricity). Also, without a mobile phone communication allowance, communication with others in the scheme becomes difficult (IDI04, Enrolment officer)*

Sixth, there were no clear monitoring and evaluation frameworks for CHF activities in any of the districts. The format of various reports was not standardized, thus making it impossible for CHF officers and EOs to report in the same manner. When it came to resource utilization, a recurring problem was reported in almost all of the districts: the lack of inventory of the equipment issued to the CHF officers and enrolment officers.

#### Factors influencing heterogeneity in performance across adoption and fidelity indicators and districts

From the survey data we found individual factors that had association with implementation outcomes. These included: occupation having a statistical significant association with adoption of job description (*p* = 0.001) and working in the scheme for more than 20 months had a marginal statistical significant association with FOI (*p* = 0.04). Moreover, FOI of components were influenced by individual factors, but only years of stay in the district had a positive statistical significant influence on reporting supply of materials (see Table 5).

The qualitative findings on factors that could explain the observed heterogeneity in performance across indicators but also across districts revealed a group of factors organized as: (i) organizational factors; (ii) socio-economic and political factors; and (iii) cognitive factors. These factors are also summarized in table (Table 7) and explained below.

**Table 7:** Factors influencing heterogeneity in implementation outcome of the CHF Iliyoboreshwa

Factors	Sub-categories	Descriptions by selected quotes
Organizational factors	Reward system: the reward system adopted by a given district to its workforce had impact to motivation and turnover of the workforce	“Commission is paid per number of households enrolled, whereby in this district the EO gets 10% commission … ….”
	Accountability in the system: the redesigned CHF workforce being mainly part-time and had other duties (main job)	“we decided to replace teachers by local people because teachers were underperforming due to heavy workload
	Availability of resources: Availability of human workforce, materials for enrolment and management of the CHF procedures, financial capacity	The scarcity of health workers and health facilities is the biggest challenge. In this district, we have only four health centres and twenty nine dispensaries instead of having twenty health centres and one hundred and three dispensaries.
	Leadership engagement and team work: some districts displayed good team work spirit from top leadership to village level actors	If all leaders in the district speak the same language (cooperate) in ensuring the community members enrol in the scheme and get health services, the performance of the scheme will improve
	Facilitation strategies: The variation in facilitation strategies per district, mainly rely on resource capacity, innovations and commitment	We use “gulio la afya” [open market for health services] strategy to promote the redesigned CHF
	Effective communication channels: The speed and effectiveness of communication between leadership and implementation teams	For example, we use WhatsApp group for communication with CHF officer and enrolment officers but also receive, feedback from CHF members through mobile phone short messages
	Number of stakeholders involved in implementation: Engagement of wider stakeholders based on their experiences with the community environment and processes, such as civil society organizations, community based leaders and influential community members	D7 has gone up until the eighth and ninth month, after the coming-projects of TASAF, where they have started the implementation of their activities. For instance we have observed that enrolment in D6 has improved very quickly because the citizens are empowered by TASAF projects
Socioeconomic and political factors	Budget gaps in local governments: Variation of sources of financing, especially, the district own source collections. Some districts are better than others	Some districts use the collected money/ own funds to buy CHF cards for the poor people who cannot afford to buy CHF cards.
	Political leaders engagement: Engagement of district commissioners, regional commissioners, members of parliaments and councillors	Political will is required to make CHF a success, the previous CHF only focused on technocrats. Political leaders are close to the community and are listened by the community. In my district, CHF is a permanent agenda.
Cognitive factors	Knowledge: knowledge about insurance concepts and the operations of the scheme among the community members as well as implementers	“We have decided to recruit volunteers to support the district CHF officers to enter and manage data in the IMIS because of inadequate computer literacy which delays timely collection of the data”
	Attitudes: Attitudes to the scheme by community members, leaders and implementation teams	“We have not so far claimed any funds from the nearby districts whose clients have been attended by us because we do think that we all belong to the government and we should not claim funds from the other government facilities
	Motivation: Motivated leaders and implementers as key to success of the scheme operations	We implement the redesigned CHF in district level by making sure that DC,DED and District Council Chairperson contribute and motivate the heads of departments to collaborate with CHF manager and the team though the response of these government leaders is low. (manager CHF)
	Perceptions: Perceptions related to usefulness of the scheme and the related services by the community members, the leaders and the implementation teams	People still have perceptions of the old CHF. All these people should be reached using village level sensitization teams (Fiona)
	Coping behaviours of implementers: implementers coping behaviours and innovations to successful implement the scheme procedures in adverse conditions	

##### Organizational factors

Respondents pointed to the organizational strengths and weaknesses of the districts as a driver of variation in implementation outcomes. The participants’ narratives revolved around the differences displayed by districts in the rewarding system, measures of accountability, availability of resources, leadership engagement, facilitation strategies, effectiveness of communication channels, and number of stakeholders involved.

The adoption of the redesigned CHF structures was slow in some districts due to a lack of resources and poor leadership engagement.

*“The way districts set their strategies to engage and ensure implementation and sustenance of the scheme procedures, depends on the amount of money (council budget) they have committed to the CHF”* “[IDI 3 Development partner]

*“Some district leaders directly engage in redesigned CHF implementation and also they support team work spirit of the implementation teams. For example, in D2, D5and D1 there is good cooperation between the District commissioner office, the District executive office, the councillors and together they have the same language about the Redesigned CHF “*[IDI 8, Regional secretariat member]

Some respondents indicated that a lack of leadership engagement and the unavailability of resources caused a lack of FOI to the scheme processes.

*“While implementing the scheme we make sure that the top district leaders (the DC, DED and District Council Chairperson) support the scheme activities. But the response of these leaders is still low and affects implementation of some CHF plans. [IDI 8, CHF manager]*

Similarly, FOI of program procedures was facilitated by innovative efforts and communication strategies and coupled with constant coaching of frontline implementers. For example, some districts used mainstream media (radio, TV, newspapers) and social media (such as WhatsApp) to reach the frontline implementation teams.

*“Some CHF managers use WhatsApp groups to communicate with the implementation teams but also to provide feedback to CHF clients “*[IDI 6, Development partner]

*“Implementation of “movement plans” [a supportive supervision matrix that clearly stipulates the movements of the CHF officer to support the enrolment officers] help to facilitate collection of CHF forms, repairing phones used for enrolments, coaching enrolment officers on enrolment procedures and health facilities on claiming funds from the CHF iliyoboreshwa*” [ IDI 10, CHF officer].

##### Socio-economic and political factors

Narratives from the interviews indicated that formulation of strategies to adopt, implement and sustain the CHF Iliyoboreshwa processes was dependent on the district fiscal capacity.

*“The way districts set their strategies to engage and ensure implementation and sustenance of the scheme procedures, depends on the amount of money (council budget) they have committed to the CHF”* “[IDI 3 Development partner]

It was also reported that public leaders (not elected by the citizens) such as the regional commissioner, and the district commissioners displayed a high level of political will towards the redesigned CHF. The high commitment of these political leaders to push districts to adopt the scheme structures and also adhere to scheme procedures.

*“All appointed leaders in the government take the redesigned CHF seriously, and is one of the criteria to assess their performance ““*[IDI 8, Regional secretariat member]

Participants in interviews also reported that elected leaders such as the members of the parliament and councillors had several reservations on supporting or not supporting the implementation of the scheme, as success or failure of the scheme implementation had direct impact on their future chances of being re-elected.

##### Cognitive factors

The educational levels of the implementation teams varied across districts. For instance, the enrolment officers at the beginning of the program were selected from retired civil servants or current civil servants residing in the beneficiary villages [civil servants have secondary school level of education or above], but later on, after a thorough assessment of their performance, some districts opted them out and selected members of the community to be enrolment officer. The differences in implementation outcome scores observed in the districts were reported to be influenced by implementers’ understanding of the scheme itself and the program procedures.

*“ we have decided to recruit volunteers to support the district CHF officers to enter and manage data in the insurance management information system (IMIS) because of inadequate computer literacy which delays timely collection of the data”* [IDI 2 Development partner]

FOI to scheme processes, such as the use of IMIS, was reported to be inversely related to the computer literacy of the implementation teams. Respondents revealed that some districts were supported by the HPSS project to address computer literacy issues and therefore boost performance.

*Volunteers from HPSS project were deployed in each of the districts to support the CHF management team to speed up the enrolment processes, particularly to hasten the entry and reporting of data to improve the claiming processes from the health facilities* [IDI 13 District leader].

## Discussion

This mixed methods study aimed at enhancing the understanding of the implementation of the redesigned CHF program. As such, the study provides a unique contribution to the existing literature on MHI in the Sub-Saharan Africa context by focusing on implementation rather than on impact outcomes. Overall, we found that adoption and FOI obtained from document review and in-depth interviews were higher than that reported by participants in survey. This study also indicates variations in implementation outcomes across the districts. The discrepancy in outcomes obtained in documents review and interviews with those obtained in the survey to individual implementers could be a result of the limitation of survey approach to elicit details of the constructs being assessed, which then leads to underreporting or over reporting. Heterogeneity in adoption and FOI indicators appeared to be shaped by the availability of resources (financial, materials and human capital), the extent of leadership engagement, innovative communication and facilitation strategies, the number of stakeholders involved and the skills of the implementers.

Both quantitative and qualitative findings indicated a higher adoption of processes aiming at equipping the frontline implementers, such as provision of job description and enrolment supplies than of processes aiming at building the scheme as a stable institution at the district level, such as provider-purchaser separation, functioning of the governance and management structures (CHF board and the management teams). A possible reason for this is that frontline implementers are at the intersection of the scheme processes and the beneficiary community and therefore equipping them may attract more clients to the scheme and lead to quick wins in terms of program uptake. The slow adoption of institutional building components may be explained by the fact that organisational change takes time and may also be influenced by changes in the enrolments at community level [34]. Moreover, the slow adoption of purchaser-provider separation especially by the council health management team (CHMT) members who managed the funds before the reforms could be an expression of passive resistance to health system changes introduced by the reforms in the scheme structure. The fact that the CHMT had to claim funds from the CHF management through seemingly complicated processes of filling claim forms, this might have precipitated the passive resistance. In addition, the passive resistance could be due to the complexity of the intervention processes and may need more political buy-in while implementing the scheme. Existing evidence suggests that resistance to the adoption of innovations may stem from fear of losing control [46] and resistance may be explicit but is often invisible in complex interventions such as the redesigned CHF, but it exists and can cause tremendous threats to sustainability [47, 48]. Expertise to accommodate innovations and stakeholders’ responsiveness represent important ingredients to successful uptake of interventions and their related innovations.

Our findings indicate high FOI scores for some process that could be viewed as simple such as review of CHF benefit package and setting new CHF premium, and low FOI scores for some process that are complex such as monitoring the scheme progress and supply of materials for the program. Complex processes that require several steps of execution and a multiplicity of actors in the execution process present a challenge in attaining high FOI and might require more emphasis when implementing complex public health interventions. Since complex interventions such as the redesigned CHF program address several complex components, the related small procedures could also be complex. Existing evidence, however, calls for carefulness in dealing with complexity and regularly adjusting the guidelines to facilitate smooth implementation of the interventions [49, 50].

The socio-economic and political push factors reported in the study affected the implementation outcomes from adoption through to FOI of the scheme processes. This finding is not surprising as the socioeconomic and political context is important in kickstarting the program and continuing the implementation process, as without committing resources and ensuring high political commitment many programs would fail [20, 24, 35, 46].

Organisational weaknesses manifested as poor rewards systems to frontline implementers, erratic availability of materials, and poor leadership engagement in some districts was the main drivers of heterogeneity of implementation outcomes across districts. These findings also align with existing evidence that attest to organisational factors as drivers of success or failure in implementing complex public health interventions [32, 34, 35, 51]. Similarly, the finding that implementers’ understanding of the scheme procedures influenced variations in FOI adds to the existing evidence suggesting the need to pay attention to the knowledge and skills of implementers to improve gains in the implementation outcome of the intervention [24, 25, 52].

The finding that the use of information management systems was challenged by the erratic supply of electricity and computer illiteracy among the implementers highlights the need to look at the context and address contextual deficiencies before deploying such technologies. Despite these challenges, deploying digital technologies to enhance implementation of MHI schemes and the use of social media to facilitate communication between implementers or with clients is a stride forwards to facilitate uptake of these schemes. WHO digital health intervention framework [53] provides guidance on how to deploy digital technologies to enhance the uptake of interventions and their innovations.

### Methodological consideration

In this study, we adopted an approach that builds on the synthesis of quantitative and qualitative methods and that takes into account the implementation outcomes of the districts and the drivers of heterogeneity of the observed outcomes. This approach enabled us to look at the implementation of the scheme as a whole and appreciate the role of the context in influencing implementation [25, 47, 54]. However, measuring implementation outcomes represents a difficult undertaking, and the use of a scoring system based on data extracted from a document review may fall short of highlighting all the relevant indicators and runs the risk of incomplete information. Still, this approach is valuable since it represents a concrete attempt to quantify variability in implementation processes in a real-world setting. Our qualitative study relied on key informants to provide information on the implementation of the scheme in their own settings; hence findings may be prone to social desirability bias. We attempted to minimize the possibility of this type of bias by using local research assistants and ensuring the confidentiality of all collected information. Also, the interview participants’ experiences may not represent the full range of perspectives of advisors, managers and implementers of the scheme. In addition, since some of the events had occurred long before our data collection, recalling the events perfectly could have been problematic, although we asked all questions to enhance recall.

### Policy recommendations

As the CHF Iliyoboreshwa is further scaled up in Tanzania, this study not only generates policy-relevant evidence that can be used to inform the CHF Iliyoboreshwa rollout in the coming years, but also highlights lessons that are transferable to MHI implementation in other similar settings. Findings from this study generate the following policy recommendations. First, the institutionalization of the CHF Iliyoboreshwa at the district level should receive much attention, while the implementation processes at the community level should also be strengthened. Stakeholders supporting districts to strengthen the implementation of the scheme should equally support activities aiming at improving governance and leadership competences related to the scheme implementation processes. Second, as the uptake of the CHF Iliyoboreshwa becomes high, further gains may be achieved by the development of sustainability strategies, such as explicitly integrating the CHF Iliyoboreshwa into the Council strategic plans or having separate CHF strategic plans. Third, this study and existing evidence [55, 56] suggest that implementers with better knowledge and skills on the program processes are likely to translate into more gains for the program (when combined with other inputs and efforts, such as the availability of materials for the program). Bringing onboard managers and implementers with training in insurance, social protection, implementation studies, and social and community development studies may help boost the implementation outcomes of the interventions. Fourth, while focusing on improving monetary incentives and supply of materials to the implementers (“the hygiene factors”), a focus on periodic refresher trainings, positive feedbacks and technical backstopping ( “the motivating factors”) should be a top priority [57, 58].

## Conclusion

The study presents an innovative effort to explain heterogeneity of implementation outcomes across the districts implementing the CHF Iliyoboreshwa in Tanzania. The study demonstrates the importance of assessing the implementation processes in the light of detecting implementation weaknesses that could be adjusted during program implementation or as the interventions are rolled out to other settings. The results from this study suggest that implementation outcomes and factors shaping these outcomes can be assessed at a higher level of implementation (such as the district level) because it is possible to obtain quantitative outcome scores and explain the potential sources of heterogeneity of the scores by interviewing individuals involved in the implementation or supporting the implementation. Results further suggest that the knowledge and skills of the individual implementer as well as contextual factors influence the implementation outcomes of the scheme. Therefore, improving individual implementer capability and addressing organisational, socio-economic and political factors could improve gains in the implementation outcomes.

## Supplementary Information

**Additional file 1. **GRAMMS framework—Checklist of items that should be included in reports of ***mixed methods studies.***

**Additional file 2.** District level Questionnaire.

**Additional file 3.** Scoring criteria for document review t for implementation process variables.

**Additional file 4.** In-depth interview- District stakeholders.

## Data Availability

The datasets used and/or analyzed during the current study are available from the corresponding author on reasonable request.

## Abbreviations

CCHP: Comprehensive council health plan
CHF: Community health fund
CHMT: Council health management team
D: District
DC: District commissioner
DED: District executive director
EO: Enrolment officer
FOI: Fidelity of implementation
HPSS: Health promotion and system strengthening
IDI: In-depth interview
IMIS: Insurance management information system
LMIC: Low and middle income countries
MHI: Micro health insurance
MTEF: Medium term expenditure framework
SOP: Standard operating procedures
TOI: Theory of intervention
UHC: Universal health coverage
