# Supplementary material for: Factors influencing variation in implementation outcomes of the redesigned community health fund in the Dodoma region of Tanzania: a mixed-methods study
Source: BMC Public Health. 2021 Jan 2;21:1. doi: 10.1186/s12889-020-10013-y (PMC7777388; doi:10.1186/s12889-020-10013-y)
Supplement: Supplementary file 1 — Additional file 1. GRAMMS framework—Checklist of items that should be included in reports of mixed methods studies. [file 12889_2020_10013_MOESM1_ESM.doc]

GRAMMS framework—Checklist of items that should be included in reports of ***mixed methods studies***

| Item No | Recommendation | Reported on page |
| --- | --- | --- |
| 1 | Describe the justification for using a mixed methods approach to the research question | 7 |
| 2 | Describe the design in terms of the purpose, priority and sequence of methods | 8 |
| 3 | Describe each method in terms of sampling, data collection and analysis | 8 |
| 4 | Describe where integration has occurred, how it has occurred and who has participated in it | 14 |
| 5 | Describe any limitation of one method associated with the presence of the other method | 26 |
| 6 | Describe any insights gained from mixing or integrating methods | 14 |
