# Supplementary material for: Factors influencing variation in implementation outcomes of the redesigned community health fund in the Dodoma region of Tanzania: a mixed-methods study
Source: BMC Public Health. 2021 Jan 2;21:1. doi: 10.1186/s12889-020-10013-y (PMC7777388; doi:10.1186/s12889-020-10013-y)
Supplement: Supplementary file 2 — Additional file 2. District level Questionnaire. [file 12889_2020_10013_MOESM2_ESM.doc]

Additional file 2: District level Questionnaire

**FACTORS AFFECTING ADOPTION, IMPLEMENTATION FIDELITY, AND SUSTAINABILITY OF RE-DESIGNED COMMUNITY HEALTH FUND PROGRAM: A MIXED METHODS STUDY IN DODOMA REGION OF TANZANIA.**

**QUESTIONNAIRE TO DISTRICT LEVEL IMPLEMENTATION TEAMS**

“Thank you for agreeing to participate in this survey. My name is **[insert your name].** I work for / on behalf of the HPSS project and University of Heidelberg, Germany). We are talking to implementers of the Redesigned CHF scheme in an effort to find out more about the implementation process of the scheme. Your contribution will be of great importance to us.

The interview will last about [**insert a tested time for Swahili interviews**].

There is no right or wrong answers to the questions; we would just like to learn about your personal thoughts and attitudes. If you don’t understand a question, please tell me, and you can add further information at any stage. Your answers will, of course, be kept confidential. Your personal responses will be seen by only a very few of my colleagues and your name will not be used in relation to the answers you give.

Place of interview……………………………… District…………………………Village…………………

Interviewers code *|__|__|*

Date: |__|__|. |__|__|. |__|__|__|__|

STARTING TIME : HOUR___________________MINUTES__________________

END TIME : HOUR _______________________MINUTES ________________

Checked by supervisor |____|1. Yes 2. No at ………. (Time)Date……………………….

DEMOGRAPHIC INFORMATION

SOCIO-DEMOGRAPHIC INFORMATION

1. Age:________ (years)
2. Sex 1. Male 2. Female |____|
3. Which job category most accurately describes you

1. VCHF officer 2. CHF manager 3. CHF accountant 4.CHF medical advisor 5.District medical officer 6. District treasurer.7. District planning officer 8. District community development officer 9. District health secretary 10. Others (please mention) |____|

1. Which section /department do you work in primarily? / Mention ………………………………….
2. How long have you worked in the program? …………..months
3. How long have you worked in this district? (years)
4. Marital status

1. Single 2.Married 3.Cohabiting 4.widow 5.others (Mention)…|____|

1. Level of education

1. Primary 2.Secondary 3 Colleges and University 4 Others ……. (Mention) |____|

9. Total number of years spent in schooling -------- (years) ______________ (years)

ADOPTION OF THE REDESIGNED CHF

10. Which of the following CHF (and related) structures are present in this village?

| A | CHF village meetings | 1.Agree 2.Disagree  3.Uncertain | **|____|** |
| --- | --- | --- | --- |
| B | Enrolment officers | 1.Agree 2.Disagree  3.Uncertain | **|____|** |
| C | CHF mobilizers | 1.Agree 2.Disagree  3.Uncertain | **|____|** |
| D | Others ( specify) | 1.Agree 2.Disagree  3.Uncertain | **|____|** |

11. Which of the following CHF manuals have you ever seen while performing your daily duties in the Redesigned CHF program?

| A | CHF standard operating procedure Manual | 1.Agree 2.Disagree  3.Uncertain | **|____|** |
| --- | --- | --- | --- |
| B | CHF financial guideline | 1.Agree 2.Disagree  3.Uncertain | **|____|** |
| C | CHF feedback forms | 1.Agree 2.Disagree  3.Uncertain | **|____|** |
| D | CHF policy guidelines | 1.Agree 2.Disagree  3.Uncertain | **|____|** |
| E | Others ( specify) | 1.Agree 2.Disagree  3.Uncertain | **|____|** |

12 .Which of the following CHF manuals do you use in your daily activities of implementing the Redesigned CHF?

| A | CHF standard operating Manual | 1.Agree 2.Disagree  3.Uncertain | **|____|** |
| --- | --- | --- | --- |
| B | CHF financial guideline | 1.Agree 2.Disagree  3.Uncertain | **|____|** |
| C | CHF feedback forms | 1.Agree 2.Disagree  3.Uncertain | **|____|** |
| D | CHF policy guidelines | 1.Agree 2.Disagree  3.Uncertain | **|____|** |
| E | Others ( specify) | 1.Agree 2.Disagree  3.Uncertain | **|____|** |

13. Do you have a job description that explains your role in the CHF operations in this village?

1Yes 2. No [go to 25) |____|

14. If yes, what are the main functions described in your job description?

i………………………………………………………………………

ii…………………………………………………………………………

15. Which functions do you actually perform in the course of implementing the Redesigned CHF program

i……………………………………………………………………………………

ii……………………………………………………………………………………

IMPLEMENTATION FIDELITY OF CHF OPERATIONS

16. Were you trained before the implementation of the Redesigned CHF?

1.Yes 2.No if Yes go to 28 |____|

17. How did you learn about the CHF enrolment procedures?

…………………………………………………………………………..

18. Which of the following practices happen in this village in terms of CHF implementation?

| A | The VEO coordinates mobilization and enrolment | 1.Agree 2.Disagree  3.Uncertain | |____| |
| --- | --- | --- | --- |
| B | The VEO handles CHF membership procedures | 1.Agree 2.Disagree  3.Uncertain | |____| |
| C | The VEO collects feedback from CHF patients and submits to the CHF manager | 1.Agree 2.Disagree  3.Uncertain | |____| |
| D | The Enrolment officer collects contributions from CHF members | 1.Agree 2.Disagree  3.Uncertain | |____| |
| E | The Enrolment officer registers households by filling out enrolment and renewal forms | 1.Agree 2.Disagree  3.Uncertain | |____| |
| F | The Enrolment officer mobilizes people to join CHF | 1.Agree 2.Disagree  3.Uncertain | |____| |

19. Which of the following statement describe actually what happens in this village with regards to materials (supply and use) of the CHF program?

| A | There has never been stock outs of enrolment materials ( package) | 1.Agree 2.Disagree  3.Uncertain | |____| |
| --- | --- | --- | --- |
| B | Enrolment materials have been timely supplied to enrolment officers | 1.Agree 2.Disagree  3.Uncertain | |____| |
| C | Enrolment materials are timely maintained after being defective | 1.Agree 2.Disagree  3.Uncertain | |____| |
| D | Enrolment officers get problems in using mobile phones | 1.Agree 2.Disagree  3.Uncertain | |____| |

20. Which of the following activities are actually happen to support you in the course of implementing the Redesigned CHF?

| A | Monthly supportive supervision | 1.Agree 2.Disagree  3.Uncertain | |____| |
| --- | --- | --- | --- |
| B | Periodic refresher trainings | 1.Agree 2.Disagree  3.Uncertain | |____| |
| C | Onsite continuous coaching by CHF officers | 1.Agree 2.Disagree  3.Uncertain | |____| |
| D | Frequent consultation over the phone in case of problems in implementing CHF processes | 1.Agree 2.Disagree  3.Uncertain | |____| |

21. Which of the following statement describes what happens with remuneration of village level CHF actors?

| A | The district council provides financial incentives to enrolment officers | 1.Agree 2.Disagree  3.Uncertain | |____| |
| --- | --- | --- | --- |
| B | The district provides no-financial incentives to enrolment officers | 1.Agree 2.Disagree  3.Uncertain | |____| |
| C | There are complaints by enrolment officers on the remuneration they get | 1.Agree 2.Disagree  3.Uncertain | |____| |
| D | Remuneration for trainings and refresher trainings are provided without delays | 1.Agree 2.Disagree  3.Uncertain | |____| |

22. What improvements have been implemented with regards to CHF benefit package?

| A | Membership fee has been changed to reflect community ability to pay | 1.Agree 2.Disagree  3.Uncertain | |____| |
| --- | --- | --- | --- |
| B | The district council provides membership cards to poor households ( individuals) | 1.Agree 2.Disagree  3.Uncertain | |____| |
| C | CHF members can be treated at any health facility in the district | 1.Agree 2.Disagree  3.Uncertain | |____| |
| D | CHF members are not charged extra money upon admission at the district hospital | 1.Agree 2.Disagree  3.Uncertain | |____| |
| E | CHF members are provided with information ( feedback) about the availability of medicines in the nearby health facility | 1.Agree 2.Disagree  3.Uncertain | |____| |
| F | Others ( specify) | 1.Agree 2.Disagree  3.Uncertain | |____| |

23. Which of the following activities (processes) actually take place in this village?

| A | CHF is a permanent agenda in all village meetings | 1.Agree 2.Disagree  3.Uncertain | |____| |
| --- | --- | --- | --- |
| B | Local radio stations have been broadcasting CHF spot messages | 1.Agree 2.Disagree  3.Uncertain | |____| |
| C | Village level leaders (Chairperson, VEO, ten cell leaders) have been frontline in sensitizing communities to enrol in CHF | 1.Agree 2.Disagree  3.Uncertain | |____| |
| D | Village chairpersons support CHF activities in their villages | 1.Agree 2.Disagree  3.Uncertain | |____| |

24. Which of the following meetings have you attended in the last months?

| A | Village CHF meeting | 1.Agree 2.Disagree  3.Uncertain | |____| |
| --- | --- | --- | --- |
| B | Claims committee meeting | 1.Agree 2.Disagree  3.Uncertain | |____| |
| C | CHF sensitization meeting | 1.Agree 2.Disagree  3.Uncertain | |____| |
| D | Meeting with the CHF officer | 1.Agree 2.Disagree  3.Uncertain | |____| |
| E | refresher training | 1.Agree 2.Disagree  3.Uncertain | |____| |
| F | Others ( specify) | 1.Agree 2.Disagree  3.Uncertain | |____| |
| G | Not attended the meeting | 1.Agree 2.Disagree  3.Uncertain | |____| |

25. If 34 g, Why did you miss to participate?.................................................

26. Who is responsible for active mobilization of the community to join CHF?

| A | Village chairperson | 1.Agree 2.Disagree  3.Uncertain | |____| |
| --- | --- | --- | --- |
| B | Ten cell leader | 1.Agree 2.Disagree  3.Uncertain | |____| |
| C | Enrolment officer | 1.Agree 2.Disagree  3.Uncertain | |____| |
| D | Village executive officer | 1.Agree 2.Disagree  3.Uncertain | |____| |
| E | Councillor | 1.Agree 2.Disagree  3.Uncertain | |____| |
| F | Others (specify)………………………….. | 1.Agree 2.Disagree  3.Uncertain | |____| |

27. Which of the following CHF operations are problematic in this village? Circle all that apply

| A | Availability of enrolment packages | 1.Agree 2.Disagree  3.Uncertain | |____| |
| --- | --- | --- | --- |
| B | Mobilizing people to enrol in CHF | 1.Agree 2.Disagree  3.Uncertain | |____| |
| C | Maintenance of phones when defective | 1.Agree 2.Disagree  3.Uncertain | |____| |
| D | operating the phone | 1.Agree 2.Disagree  3.Uncertain |  |
| E | Filling the enrolment forms | 1.Agree 2.Disagree  3.Uncertain |  |
| G | Others (specify)………………………….. | 1.Agree 2.Disagree  3.Uncertain | |____| |

28. How are poor people enrolled in CHF in this village?

| A | They receive CHF cards paid by TASAF | 1.Agree 2.Disagree  3.Uncertain | |____| |
| --- | --- | --- | --- |
| B | They receive CHF cards paid by Village government | 1.Agree 2.Disagree  3.Uncertain | |____| |
| C | They receive CHF cards paid by district council | 1.Agree 2.Disagree  3.Uncertain | |____| |
| D | NGOs / local groups pay for them | 1.Agree 2.Disagree  3.Uncertain | |____| |
| E | Good Samaritans pay for them | 1.Agree 2.Disagree  3.Uncertain | |____| |
| F | They receive exemptions when they visit health facilities | 1.Agree 2.Disagree  3.Uncertain | |____| |
| G | No available mechanism to enrol poor people | 1.Agree 2.Disagree  3.Uncertain | |____| |
| H | Others (specify) | 1.Agree 2.Disagree  3.Uncertain | |____| |

29. Were there any changes (additions, deletions, modifications) of some procedures stipulated in CHF-SOP/ job description in order to implement smoothly the Redesigned CHF ? 1. Yes 2.No |____|

30. Mention the changes that were made to facilitate CHF implementation in this district

……………………………………………………………………………………………………………

31. How many times did the CHF officer visit this village in the past six months?....................

32. How many times did the CHF officer visit this village in the past one month? …………………..

33. What activities were conducted when the CHF officer visited this village?

……………………………………………………………………………………………………………

34. Have you ever received instant incentive payments in the course of accomplishing CHF activities?

1.Yes 2.No

35. Why did you receive this payment?

|____|

36 If not, how long does it take to receive incentives?..........................(days)

37. When was the last time you received this payment?.....................

38. Do you receive any non- financial incentives?

1.Yes 2.No |____|

39. Which ones? (specify) ……………………………………..

40. How long does it take to enter the data in IMIS system from the time enrolment takes place? ………………….(days)

MODERATING FACTORS OF CHF OPERATIONS

41. What are the top three factors that have been negatively affecting CHF implementation in this village? Tick only three

| a | Lack of commitment of village leaders | |____| |
| --- | --- | --- |
| b | Complexity of the procedures of enrolling clients | |____| |
| c | Negative attitudes of the community with regards to CHF | |____| |
| d | Lack of team work among implementers at village level | |____| |
| e | The councillor does not support CHF activities | |____| |
| f | Lack of team work among implementation teams | |____| |

42. What are the top three factors that have been facilitating the implementation of CHF in this village? Tick only three

| a | Enrolment officers get good support from the district | |____| |
| --- | --- | --- |
| b | Many people are enthusiastic to join CHF sensitization meetings | |____| |
| c | There is team work in implementing the CHF | |____| |
| d | Availability of CHF guidelines and manuals | |____| |
| e | The premium is affordable by most people | |____| |
| f | The quality of health care services in the nearby facility is acceptably high | |____| |
| g | The community is supportive to the enrolment officer activities | |____| |
| h | The community understands well the Redesigned CHF | |____| |
| i | Influential people( such as religious leaders, political party leaders, traditional leaders) are not supportive to CHF | |____| |

43. Which of the following are true about CHF implementation in this village?

| A | The village authority takes CHF a compulsory agenda in all village meetings | 1.Agree 2.Disagree  3.Uncertain | |____| |
| --- | --- | --- | --- |
| B | There is joint meetings between enrolment officers and the VEO with regards to CHF | 1.Agree 2.Disagree  3.Uncertain | |____| |

**44. Suggestions/Comments:** Please provide any comments and/or suggestions that can make CHF implementation be improved in this district ………………

***Thank you for your participation***
