# Supplementary material for: Factors influencing variation in implementation outcomes of the redesigned community health fund in the Dodoma region of Tanzania: a mixed-methods study
Source: BMC Public Health. 2021 Jan 2;21:1. doi: 10.1186/s12889-020-10013-y (PMC7777388; doi:10.1186/s12889-020-10013-y)
Supplement: Supplementary file 3 — Additional file 3. Scoring criteria for document review t for implementation process variables. [file 12889_2020_10013_MOESM3_ESM.doc]

Additional file 3: Scoring criteria for document review t for implementation process variables

| **Variable** | **Item** | **Item score (%)** |
| --- | --- | --- |
| Adoption | CHF office location |  |
|  | CHF office located in a separate unit | 100 |
|  | CHF office located in another department of the district council | 50 |
|  | CHF office located in the health department | 50 |
|  | Presence of a Memorandum of understanding with HPSS project |  |
|  | Presence of signed MOU | 100 |
|  | Presence of a contract between CHF management teams and CHMT |  |
|  | Presence of contract (initial process) | 50 |
|  | Presence of signed Contract | 100 |
|  | Presence of job description of CHF staff |  |
|  | Presence of job description | 50 |
|  | Job description given to staff | 100 |
|  | Presence of functioning CHF management teams |  |
|  | Presence of CHF management teams | 50 |
|  | CHF management teams meeting regularly (evidenced by meeting minutes) | 100 |
|  | Presence of functioning CHF boards |  |
|  | Presence of CHF board | 50 |
|  | CHF boards meeting regularly (evidenced by meeting minutes) | 100 |
| Fidelity of implementation | Training of the CHF structures ( CHF board, CHF management teams and EOs) |  |
|  | Presence of training reports for all CHF structures | 100 |
|  | Presence of training reports to new recruited staff | 50 |
|  | Presence of reports of staff who attended refresher trainings | 50 |
|  | Materials for the program |  |
|  | Presence of completely filled inventory for program materials | 100 |
|  | Presence of partially filled inventory for program materials | 50 |
|  | Remuneration of staff and health facilities |  |
|  | Presence of evidences for remuneration of health facilities | 50 |
|  | Presence of evidences for remuneration of staff (Eos, CHF officers and other staff) | 50 |
|  | Monitoring progress of the program implementation |  |
|  | Presence of supportive supervision reports | 30 |
|  | Presence of Movement plan reports | 30 |
|  | Presence of meeting minutes | 40 |
|  | Addressing the benefit package |  |
|  | Reports on enrolment of poor people | 30 |
|  | Presence of district budget for pro-poor enrolment | 30 |
|  | Proof of participatory review of the premium and benefit package as evidenced by minutes from WDCs and District Full council meetings | 40 |
|  | Quality of health care |  |
|  | Availability of reports of medicines availability | 50 |
|  | Reports are periodic (monthly, quarterly or semi-annual) | 50 |
|  | Promotion and sales forces |  |
|  | List of NGOs, local groups that participates in CHF activities | 20 |
|  | Reports of participation of local groups in promotion activities | 20 |
|  | Reports of mass media campaigns implemented in the district | 20 |
|  | Reports on participation of local leaders in campaigns |  |
|  | Reports of mass campaigns implemented in villages | 20 |
| Sustainability | Strategy to sustain CHF processes |  |
|  | Presence of strategic plan (3, 5 or 10 years) | 50 |
|  | Presence of CHF activities in district plans | 50 |
|  | Maintenance of program process |  |
|  | Presence of health facility accounts | 20 |
|  | Presence of record management policy | 20 |
|  | Presence of physical asset management policy | 20 |
|  | Presence of CHF monitoring framework | 40 |
