# Supplementary material for: Factors influencing variation in implementation outcomes of the redesigned community health fund in the Dodoma region of Tanzania: a mixed-methods study
Source: BMC Public Health. 2021 Jan 2;21:1. doi: 10.1186/s12889-020-10013-y (PMC7777388; doi:10.1186/s12889-020-10013-y)
Supplement: Supplementary file 4 — Additional file 4. In-depth interview- District stakeholders. [file 12889_2020_10013_MOESM4_ESM.doc]

Additional file 4: In-depth interview- District stakeholders

FACTORS AFFECTING ADOPTION, IMPLEMENTATION FIDELITY, AND SUSTAINABILITY OF RE-DESIGNATED COMMUNITY HEALTH FUND PROGRAM: A MIXED METHODS STUDY IN DODOMA REGION OF TANZANIA.

| Number of interview |  | |  |  | | |
| --- | --- | --- | --- | --- | --- | --- |
| Interviewer name | | |  | | | |
| Date | | | date | Month | year | |
| Time started | Hour | Minutes | Time of finishing the interview | | Time | minutes |

Greetings! My name is ………………….. I am a researcher (research assistant) from HPSS project and Heidelberg University. Thank you for taking the time to participate in this interview. This interview is being conducted to get your views on the implementation of the Redesigned CHF program. The findings from this research will help improve the way the CHF program is supposed to be implemented and also provide recommendations for scale up to other places. I am especially interested in any problems you are aware about this issue, experiences and recommendations for further improvements.

"If it is okay with you, I will be tape recording our conversation. The purpose of this is to get all the details but at the same time be able to carry on an attentive conversation with you. Your opinions and experiences are important for us. Your answers will be confidential and not traced to you as person.

If you agree to this interview and the tape recording, please sign this consent form." *[give the consent form to the participant]*

I'm now going to ask you some questions that I would like you to answer to the best of your ability. If you do not know the answer, please say so. If you are uncomfortable with a question, you do not have to answer it if you wish

1. Please describe your role and position in this district

Probes: *What is your formal position?*

*What are your duties and responsibilities?*

*What do you do in daily practice?*

1. What is your role and position in the implementation of CHF?
2. Can you describe the time you have been working with this program?
3. What was your knowledge on CHF before introduction of the Redesigned CHF?
4. How did you first hear about the Redesigned CHF?

Probe: *How were you introduced to the program?*

*Who introduced the program?*

1. How was the concept of a Redesigned CHF explained to you?

Probes: *How were you involved in the initial steps that adopted the scheme?*

*What was your role in the adoption of the scheme?*

1. What initial decisions needed to be made in order to start the new scheme?
2. What motivated the district to adopt this new scheme?

Probe: Any dissatisfaction with the old CHF structure?

1. How systems are in place to ensure effective implementation of the scheme?

Probe; *Availability of resources (Financial, human, materials)*

*How is the district council engaged in implementation of CHF?*

1. What strategies do you use to attract people to join CHF?

Are you doing it differently compared to 3 years ago?

1. How do services / activities of the Redesigned CHF cater for different groups in the community?

Probes*: How about poor households?*

*How do you identify the poor?*

*How about people who stay far from district headquarters/ village headquarters?*

1. How do you engage the community in the course of CHF implementation?

Probes: *How about feedback of the CHF implementation to the community?*

*How about participation in village CHF meetings?*

1. How do you engage enrolment officers and health care providers to maximize enrolment in CHF?

Probe: *Can you describe what activities are carried by enrolment officers and health care providers?*

1. How is the progress of the implementation of the program monitored?
2. In your opinion, can you say that you implement what you planned (or as stipulated in CHF SOP) or you are deviating / modifying depending on the circumstances?

Probes: *(If applied modifications), can you describe what you modified?*

*Were there no other alternatives than applying modifications?*

- *With whom did you discuss such changes?*
- *What are the things that are happening in the CHF now that you didn’t plan for?*

1. What are the major challenges / constraints to the implementation of Redesigned CHF?

Probes: *Who/ what hinders its implementation*

*How about responsiveness of various stakeholders*

*Are there any conflicts in terms of managing day to day operations of CHF (such as management of CHF Funds)*

*How about the understanding (description) of the design of the scheme?*

*How about economic and political situations?*

1. What are the major facilitators to the implementation of CHF?
2. What can you say about the reactions of stakeholders about the Redesigned CHF?

Probes: *How about district leaders*

*How about health care providers*

*How about religious leaders*

*How about the general community*

1. Describe the support you get in the process of implementing the Redesigned CHF

Probes: *Who provides support?*

*Which support do you exactly get?*

*Any support you get but not needed?*

Follow up questions to some key informants

| Participant | Questions |
| --- | --- |
| District Medical officer | 19a. What is your experience on the way health facilities claim funds from the Redesigned CHF ?  19b. How do the health facilities actually claim money? ? are there any challenges?   - which procedures are in place to make sure the claims are correct? - How about the correctness of the diagnoses? - what happens after getting the claimed funds?   19c. How about availability of medical supplies in health facilities?  19d. what is your comment about the quality of health care provided in health facilities?  How about the care of CHF members? |
| CHF manager | 19e. Which guidelines on CHF are available here?   - How about their use in daily routines? can you provide an example - How about CHF movement plans? - Is there any sustainable strategy to maintain the CHF processes?   219f what are the CHF components for 2014/2015?  (*Can I get a copy of the current budget?)* |
| CHF accountant | 19g What is your experience on the way health facilities claim CHF funds?  19h. What are the challenges about claiming and reimbursements for health facilities?  19i. what are your experiences about collection of CHF funds from enrolment officers?  19j How about claims of matching grants from the national level? ?  Are there any clear differences from the old CHF? |

1. What are your general feelings with regards to re-CHF implementation?
2. What would you recommend to do differently if you were to restart today with the experience you have gathered in the mean time?
3. What would you suggest to be done differently to improve performance and sustainability if this program is to be implemented in other regions?
4. Is there anything else we haven’t discussed yet that you think is important for CHF implementation

**That concludes our interview**

***Thank you so much for sharing your thoughts and opinion with us***
